# Characterization and Filtration Efficiency of Sustainable PLA Fibers Obtained via a Hybrid 3D-Printed/Electrospinning Technique

**DOI:** 10.3390/ma14226766

**Published:** 2021-11-10

**Authors:** Mattia Pierpaoli, Chiara Giosuè, Natalia Czerwińska, Michał Rycewicz, Aleksandra Wieloszyńska, Robert Bogdanowicz, Maria Letizia Ruello

**Affiliations:** 1 Department of Metrology and Optoelectronics, Faculty of Electronics, Telecommunication and Informatics, Gdańsk University of Technology, 80-233 Gdańsk, Poland; michal.rycewicz@pg.edu.pl (M.R.); aleksandra.wieloszynska@pg.edu.pl (A.W.); robbogda@pg.edu.pl (R.B.); 2 INSTM Research Unit, Department of Materials, Environmental Sciences and Urban Planning (SIMAU), Università Politecnica Delle Marche, 60131 Ancona, Italy; n.czerwinska@pm.univpm.it (N.C.); m.l.ruello@univpm.it (M.L.R.)

**Keywords:** electrospinning, filtering materials, PLA, filtration efficiency, fibers, biodegradable

## Abstract

The enormous world demand for personal protective equipment to face the current SARS-CoV-2 epidemic has revealed two main weaknesses. On one hand, centralized production led to an initial shortage of respirators; on the other hand, the world demand for single-use equipment has had a direct and inevitable effect on the environment. Polylactide (PLA) is a biodegradable, biocompatible, and renewable thermoplastic polyester, mainly derived from corn starch. Electrospinning is an established and reproducible method to obtain nano- and microfibrous materials with a simple apparatus, characterized by high air filtration efficiencies. In the present work, we designed and optimized an open-source electrospinning setup, easily realizable with a 3D printer and using components widely available, for the delocalized production of an efficient and sustainable particulate matter filter. Filters were realized on 3D-printed PLA support, on which PLA fibers were subsequently electrospun. NaCl aerosol filtration tests exhibited an efficiency greater than 95% for aerosol having an equivalent diameter greater than 0.3 μm and a fiber diameter comparable to the commercially available FFP2 melt-blown face mask. The particulate entrapped by the filters when operating in real environments (indoors, outdoors, and working scenario) was also investigated, as well as the amount of heavy metals potentially released into the environment after filtration activity.

## 1. Introduction

The current SARS-CoV-2 virus, which causes the COVID-19 disease, is transmitted by droplets (e.g., coughing, speaking, sneezing, and singing) through close contact with an infected person [1,2]. These particles are divided into respiratory aerosols (diameter > 5 µm) and droplet nuclei (diameter ≤ 5 µm) [3] which are a residue of dried respiratory aerosols as a result of evaporation. Smaller aerosols penetrate and linger in the lower parts of the respiratory system, while larger aerosols are deposited in the upper parts [4]. Therefore, in order to minimize the risk of contamination, it is advisable to wear masks or respirators. In addition to the current pandemic, filtering masks are also used in industrial workplaces (outdoors and indoors) and are very common in countries where the air quality is very poor, to protect the respiratory tract from pollutions. Particle pollutants, which are divided into two groups—PM_10_ (particles with an aerodynamic diameter less than 10 μm) and PM_2.5_ (particles with an aerodynamic diameter less than 2.5 μm), come from various sources, both internal and external, and consist of dust, pollen, mold, heavy metals, organic compounds, and combustion particles. For example, industrial sources include coal burning, metal processing, petroleum combustion, biomass burning, and production processes [5]. Other sources of pollution include transport, stationary combustion sources, and natural sources such as volcanic eruptions [6].

National and international organizations define the standards to classify mask filtration efficiency according to the percentage of withheld particles having a specific nature and characteristic diameters. According to the European standard EN-149: 2001 [7], three classes of masks are defined—FFP1, FFP2, and FFP3. The filtration efficiency for FFP1 is not less than 80%, that for FFP2 is not less than 94%, and that for FFP3 has the greatest filtering ability with 99% (tested with sodium chloride and paraffin oil aerosols). The relevant document for the certification of particulate respirators in the United States is that published by the National Institute for Occupational Safety and Health (NIOSH), called 42 CFR Part 84. It distinguishes nine filter classes—three filter performance categories, each divided into three levels of resistance to degradation by oily aerosols. The first three categories allow a minimum filtration efficiency of 95%, 99%, or 99.97%. Filter performance is related to the capture of an aerosol that is the most penetrating, with a diameter approximately of 0.3 μm [8].

Tutorials and do-it-yourself guides on how to solve face mask shortages with easily available material have spread on the world wide web. However, the efficiency of the latter is highly questionable, and wearing such homemade solutions could lead the concerned individuals to expose themself to risk which, normally, they would not be exposed to. On the other hand, the current situation has seen an increase in activity within the online community of makers as a result of the COVID-19 emergency. The most successful stories include the production of face shields [9], the creation of an adapter to transform diving masks into continuous positive airway pressure (CPAP) masks for hospital respirators [10], and the production of ventilator valves. The production of protective masks, through 3D printing, is instead limited since it is impossible to realize an effective filter with a 3D printer.

Most disposable masks are made of polymers such as polypropylene, polyurethane, polycarbonate, polyacrylonitrile, polyethylene, polystyrene, or polyester [11], while masks with filtration classes FFP2 and FFP3 are generally made of polypropylene nonwoven fabric. The process uses the technique of melt blowing, where the melted polymer is extruded from small nozzles to form micro- and nanofibers, allowing for high filtration efficiencies. Recently, studies have shown strong evidence of the environmental impact of PPE such as disposable masks [12,13], pointing them out as a potential source of (micro-)plastic. When they get to the sea, oceans, and other bodies of water, they break down into smaller elements (some with a diameter of less than 5 mm) under the influence of various external factors. Hence, there is a need to replace nonbiodegradable materials with environmentally friendly polymers. One of the synthetic biodegradable polymers is poly(lactic acid) (PLA, [CH(CH_3_)COO]_n_), a polymer which can be produced from annually renewable resources through fermentation and polymerization processes [14,15]. Nanofibers are used, among others, in filtration processes. They owe their valuable filtration properties to their large surface area and small pores between the fibers. One of the methods of obtaining nanofibers is electrospinning, which is known for its relative simplicity [16]. A typical setup consists of a nozzle connected to a high-voltage power supply, from which the polymeric solution is extruded, and a grounded collector electrode placed at a sufficient distance from the nozzle. While the melt-blowing technique cannot be scaled down, electrospinning is a technique that allows obtaining micro- and nanofibers with limited equipment and in a modular and scalable way. Nonwoven fabrics produced with this technique have already been studied as efficient filter media for atmospheric particulate matter (PM). Few attempts have been reported to integrate electrospun nanofibers with 3D printing, both directly [17] and through a multiple-step process [18,19,20]. However, a critical step is to obtain sufficient adherence between the 3D-printed and electrospun layer, especially when different materials are used [19]. In a recent study, He and coworkers realized PLA composite material via a two-step process, by electrospinning PLA fibers over aluminum foil, followed by 3D printing over it, by gluing it to the printer heated bed [20]. The result is a transparent, self-sustaining filter, and the main advantage of their approach is mechanically enhanced substrate–fiber adhesion. One significant attempt to combine the two techniques in a single setup was done by Rivera and Hudson, whereby the extruder of a commercially available 3D printer was adapted to produce fibers by electrostatic forces [17]. This approach allowed the processes to be greatly simplified, while limiting the use of hazardous solvent and partially solving the problem of heteromaterial compatibility; on the other hand, obtaining a nanometric-size fiber diameter was challenging due to the low applied potential (7 kV) and short distance between nozzle and 3D printing/collecting plate. For this reason, decoupling the two manufacturing methods allows controlling and individually optimizing the process parameters.

Whereas, in many papers dealing with the electrospinning technique, many authors often refer to in-lab built electrospinning apparatus, few of them focused on their description or made such equipment available on the internet. Barraza et al. designed a low-cost electrospinning chamber for education purposes, using acrylic sheets and a vertical collector; however, both the syringe pump and the high-voltage power supply were obtained elsewhere [21]. A very simple electrospinning apparatus for basic research and educational purposes was designed by GaudiLabs and made available online, with benefits such as the use of minimal electronic and material equipment [22]. A more complex apparatus was built by Miller, using materials commonly available, for educational purposes [23]. A brief literature review of PLA-based electrospun fibers is reported in Table 1.

In this study, for the first time, an autonomous and inexpensive open-source electrospinning setup was designed, realized by 3D printing, and optimized, to obtain environmentally sustainable and high-efficient PLA filter composites. Filters were realized in PLA via a mixed technique of 3D printing and electrospinning, simply using a commercial PLA filament and acetone as solvent. Filtration tests were performed both in the laboratory and on a real scale to assess the effective filtration performance. The potential release of heavy metals from the filter composite into the environment, due to improper disposal, after three different typical working scenarios, was also evaluated.

We show how the 3D-printing technique can be employed to successfully realize both the electrospinning setup and the scaffold of the filter itself. The electrospinning setup was realized using widely available components and 3D-printed parts, which have been made available online. Its optimized version measures 30 × 30 cm, and it costs approximately 50 EUR (Supplementary Materials). From a “cradle to grave” perspective, we demonstrate the possibility of realizing PLA-based filters in a simple, inexpensive, and delocalized way, in addition to an evaluation of the potential impact on the environment.

## 2. Materials and Methods

### 2.1. Design and Realization of the Electrospinning Setup

The electrospinning project was realized in FreeCAD (Release 0.19) and 3D-printed (Creality Ender-3) in PLA (1.75 mm, Print-me ECOline PLA). The 3D-printed parts account for a total of approximately 14 h of printing time and 150 g of raw material (PLA filament). The full bill of materials (BOM) is reported in the Supplementary Materials, and the entire optimized electrospinning system can be realized in approximately 47 EUR with a few hours of assembly. Most of the mechanical parts can be found in a hardware store, and the electronic parts can be found online or in an electronic store. An Arduino-based microcontroller board is used to operate the system and the control is made possible through a user-friendly interface that allows regulating the syringe pump speed and its longitudinal velocity parallel to the collector. The spinning process parameters can be set up through the controller user interface. The collector cylinder was obtained from a potato-snack can. The original and .stl files to realize the electrospinning setup are available online, together with the Arduino code.

### 2.2. Fabrication of the PLA-Based Filtration Mask

Filters supports were 3D-printed using a PLA filament (1.75 mm, Print-me ECOline PLA) with a nozzle diameter of 0.4 mm, a layer thickness of 0.2 mm, a nozzle temperature of 210 °C, and a printing bed temperature of 70 °C. The electrospinning solution consisted of 14 wt.% PLA pellets (Total Corbion PLA) dissolved in acetone (min. 99%, Chempur, Poland). The solution concentration was optimized to obtain beadless fibers [31]. Alternatively, a PLA filament can be used and preferably dissolved in DCM/DMF (4:1) [14]; however, we report only the filtering medium obtained from PLA pellets in acetone due to the use of acetone as a preferable solvent for its availability and low toxicity. The preparation procedure involves the addition of the solvent to the polymer, followed by mixing, without sonication, in order to not introduce non-negligible changes to the polymer structure [32]. The collector–needle distance was fixed to 10 cm, and the syringe pump was set at 5 mL/h. The relative humidity during electrospinning was equal to 41% ± 8%. The 3D-printed mesh support was placed over aluminum foil on the collector, and, after 2 h of spinning, a total surface of approximately 0.1 m^2^ was uniformly covered. Upon completing the electrospinning process, two different sets of filters were compared: one obtained by placing another 3D-printed mesh over the electrospun fiber and the other by overlapping two single layers (labeled S-PLA and D-PLA). The sandwiched filters were put between two stainless-steel plates, secured with two clamps, and dried in a vacuum oven at 40 °C for 2 h (−60 kPa).

### 2.3. Filter Characterization

The study of the morphology of the electrospun filters was provided through a scanning electron microscope (Thermo Fisher Scientific, Phenom XL, Waltham, MA, USA) equipped with an energy dispersive X-ray analysis (EDAX) probe to classify the elemental composition of the PLA fiber and the particulate retained. The image analysis was provided using discretization of at least three images per sample and the fiber diameters of the different filters were evaluated. Scanning electron microscope (SEM) images analysis was also provided to support the filtration tests.

### 2.4. Filtration Tests

#### 2.4.1. Laboratory Test

Four different types of filters were analyzed and compared: a surgical mask (commercial), an FFP2 mask (commercial), and single- (S-PLA) and double-layer (D-PLA) electrospun PLA filter membranes. The tests were carried out in an experimental test room (3 m × 2 m × 2.5 m), which provided a controlled microclimate. To generate the aerosol, an ultrasonic air humidifier (Medisana UHW, Neuss, Germany) was used to generate monodisperse aerosol of sodium chloride (NaCl) from a 2% water solution. Three fans placed inside the test room ensured homogenization of the particles of NaCl and the recirculation of air. For sampling of indoor conditions, at a height of 1.05 m from the ground, a circular hole with a diameter of 5 cm was provided, into which a cylindrical tube of the same diameter was inserted. Firstly, the moisture was removed using a commercial sorbent; then, the filter membrane was placed at the tube end, and a fan run by a variable power source was used to ensure a controllable flow through the filter medium. At the outlet, PM distribution and concentration were measured using a portable laser scattering-based optical particle counter (OPC, GRIMM Aerosol Technik GmbH, Ainring, Germany) within the diameter range of 0.23 μm to 20 μm. The efficiency and penetration of particles were determined by measuring the concentration of the aerosol with the filter and without the filter. Three test replicates for each of the filter were carried out in order to obtain reliable results.

The pressure drop (Δ*P*) across the filter was measured using MPXV7002DP (NXP Semiconductors, Eindhoven, Netherlands) piezoresistive transducers. Face velocity was measured using a hot-wire anemometer (Testo 445) across multiple points at the flow outlet.

The filtration efficiency (η) was evaluated as follows:

(1) $$\eta =1-\frac{C\;downstream}{C\;upstream}\times 100,$$

where C*_downstream_* is the average concentration of NaCl aerosol after filtration, and C*_upstream_* is the average concentration of NaCl aerosol before filtration.

Therefore, the quality factor was provided to compare the different filtering media. Equation (2) defines the quality factor (*QF*).

(2) $$QF=\frac{-\ln\left(1-\eta \right)}{∆P},$$

where η is the filtration efficiency, and Δ*P* is the pressure drop.

The *QF* was also reported for each PM diameter efficiency.

#### 2.4.2. Real-Environment Test

Electrospun PLA filters were also tested in real environments. Three different environments were chosen: (i) an outdoor (outdoors) one and two different indoor conditions, (ii) residential (indoors residential) and (iii) industrial (indoors industrial). The outdoor environment was a suburban environment, whereas indoor environments were a civil building (indoors residential) and a productive factory (indoors industrial) where the main activity is welding. All environments were located in the same city: Urbino (Italy).

PM concentrations were measured before (inlet) and after (outlet) filtration when operating at a constant flow of 9.8 L·min^−1^, employing two low-cost laser-based optical particle counters (OPCs, Nova Fitness SDS011). The accumulated total PM mass on the filter was determined by weighing the filters using a microbalance before and after exposure and by computing the difference in weight. Filters were then oxidated at 700 °C, and the powders were extracted with nitric acid (1:10 by weight); the heavy-metal concentration of the acid solution was assessed using an inductively coupled plasma spectrophotometer (ICP-OS, Optima 8300, PerkinElmer, Waltham, MA, USA). Metal concentrations were normalized on the volume (m^3^) of filtered air and clustered into three sample environments (outdoors, indoors residential, and indoors industrial).

## 3. Results and Discussion

### 3.1. Electrospinning Setup and Mask Realization

The use of commercial proprietary equipment for electrospun fiber production often entails a significant cost, mostly related to design, production, and maintenance. However, the cut-to-the-bone equipment includes a high-voltage (low current) power supply, a syringe pump, and a rotating collector (this is optional but needed for better uniformity of the fibers). To reduce costs and promote the easiness of realization and assembly, we opted for components that can be easily found in electronic and hardware stores.

**Figure 1:**
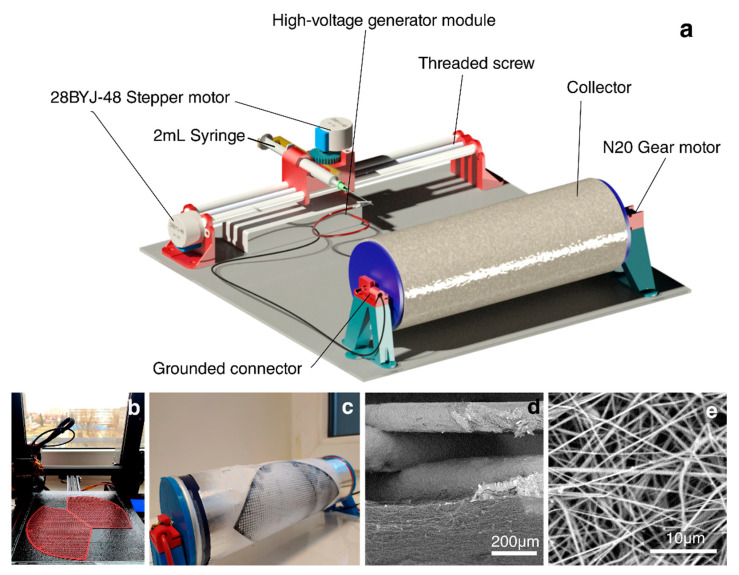
(**a**) Illustration of the 3D-printed simplified electrospinning setup; (**b**) 3D printing of the filter mask support; (**c**) electrospinning of the fibers over the 3D-printed PLA support; SEM images ((**d**): cross-section; (**e**): top-view) of electrospun nanofibers.

The model of the fully assembled device is shown in Figure 1a, and the main component assembly is reported in the Supplementary Materials (Table S1, Figures S1–S4). The printable components, bill of materials (BOM), full instructions, and programming code are publicly available in the Supplementary Materials and online. The filter support was 3D-printed in PLA (Figure 1b), placed over aluminum foil, and secured with tape to the electrospinning collector (Figure 1c). The rotary collector and the longitudinal syringe slider were introduced to achieve the target of obtaining uniformly distributed and bead-free nanofibers. Controlling the fiber diameter and average pore size is necessary to reduce the air resistance, thus enhancing the slip effect. Two of the 3D-printed and electrospun filter composite materials were overlapped, clamped, and dried at 40 °C for 2 h. Due to the material compatibility between the 3D-printed structure and the electrospun fiber, good adhesion between the two components was achieved. Figure 1d shows the cross-section of the composite, in which it is possible to observe, in the upper part, the 3D-printed layer supporting the fibrous filter. A rule of thumb is that a higher spinning voltage results in more favorable formation of fibrous membranes with a high length–diameter ratio, which improves the air filtration efficiency. An average fiber diameter of 0.48 μm was realized by electrospinning (Figure 1e), which is comparable to that found in the literature (Table 1) for filters realized with a commercially available electrospinning setup.

### 3.2. Comparison of the PLA-Based 3D-Printed/Electrospun Filtration Efficiency

#### 3.2.1. Laboratory Test Results

The peculiarity of electrospinning to produce ultrathin fiber diameters allows enhancing the aerodynamic behavior of airflow around the fiber boundaries, due to the reduced drag force. In particular, the gas flow regime across the fiber is dependent both on its diameter and on the mean free path of air molecules, and it can be described by the Knudsen number.

(3) $$Kn=\frac{2\lambda }{d_{f}},$$

where λ is the mean free path of air molecules, and *d_f_* is the diameter of the fiber.

**Figure 2:**
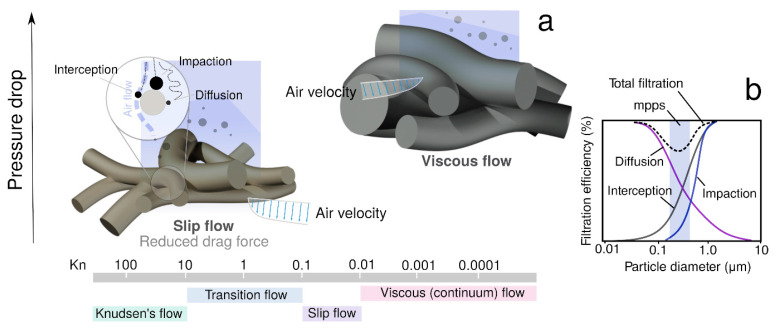
Classification of flow regimes with Knudsen number: (**a**) comparison of the flow regimes with different fiber diameters; (**b**) dependency of the filtration mechanism on the particle diameter.

The particles are captured mechanically by various mechanisms: diffusion, interception, gravitational settling, and inertial impaction. Since the fiber diameter falls within the range of hundreds of nanometers, for the PLA filter, the Knudsen number falls within the transition and slip flows (Figure 2a) [33].

For particles with diameter below 100 nm, the dominant mechanism of capture is diffusion. Brownian motion, which is the chaotic movement of particles in still air, causes the aerosol to collide with the fibers of the filter material [34]. Particles with a diameter of 0.23–0.3 μm show the lowest efficiency to be captured by filter fibers, and they are described as the most penetrating particle size (mpps) (Figure 2b). The key role in capturing particles larger than 1 μm is attributed to inertial impaction and interception. Inertial impact changes the direction of the particles in the air stream. In this case, as the diameter and face velocity increase, the inertia increases; thus, the particles are easier to retain by the filter medium. On the other hand, the interception mechanism occurs when a particle is moving toward the air streamline and comes in contact with the surface of the fiber.

**Figure 3:**
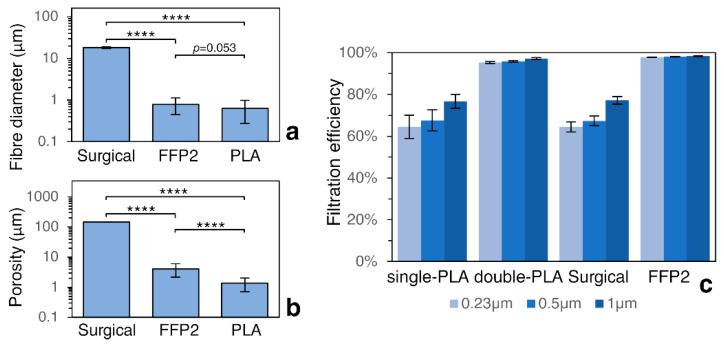
Properties of corresponding filtration media, Knudsen number regimes, flow mechanisms, and filtration efficiency: (**a**) fiber diameter; (**b**) porosity; (**c**) percentage filtration efficiency of particles with diameter 0.23 μm, 0.5 μm, and 1 μm. Significance is expressed by the *p*-value (**** *p* < 0.005).

The fiber diameter, which is a key structural parameter of filter materials, determines the filtration efficiency. Using SEM image analysis, the fiber diameter of the three filters was measured and averaged (Figure 3a). The largest value was found for the surgical mask fabric, where the average diameter was 18.19 μm, while the diameter of the obtained PLA fibers was 630 nm, and this value was similar to the diameter of FFP2 fibers (787 nm). The smallest diameter was found for electrospun PLA nanofibers (630 nm). The surgical and FFP2 mask also had a more homogeneous fiber diameter (in each layer) than the PLA filters, conferring the electrospun filter with a higher filtering capacity since the complexity of the geometry was increased (Figure 1e). The interfiber distance was considered representative of “porosity”, and the averaged values are reported in Figure 3b. PLA filters showed the lowest porosity (about 1 μm), related to the greater packing of the fibers which made the filtration medium highly efficient, but resulted in faster filter exhaustion due to higher retention of particles.

Figure 3c shows the filtration efficiency of NaCl aerosol particles with the characteristic diameters of 0.23 μm, 0.5 μm, and 1 μm through various filter media. The highest filtration efficiency, over 95% across the range of measured particles, was demonstrated by the FFP2 mask, which was comparable to the double-folded electrospun PLA filter. The S-PLA achieved a filtration efficiency similar to the surgical mask (from 60% for the smallest particles and to about 70% for the 1 μm particles). The factor that influenced the differences in filtration efficiency between D-PLA and the surgical mask was fiber diameter. In short, the filtration efficiency increases when the fiber diameter decreases. A lower penetration of the particles can be found in filters with a significantly small diameter (0.27 μm) [25] due to the increase in surface area. Multi-ayer electrospun fibers (double-PLA) had increased filtration efficiency by about 35% compared to single-layer fibers due to the increase in thickness of the filter medium. However, this came at the cost of a decrease in air permeability. Both these factors, filtering efficiency and pressure drop, affect the final value of the quality factor. Thus, the key is to design a highly effective mask suitable for everyday use for humans, while ensuring proper air circulation when inhaling and exhaling. The results reveal that double-PLA electrospun membranes exhibited similar QF values to the FFP2 (0.014 Pa^−1^ and 0.016 Pa^−1^, respectively), while the values were two-fold higher than the single-PLA and surgical mask (0.007 Pa^−1^).

#### 3.2.2. Real-Scale Test Results

**Figure 4:**
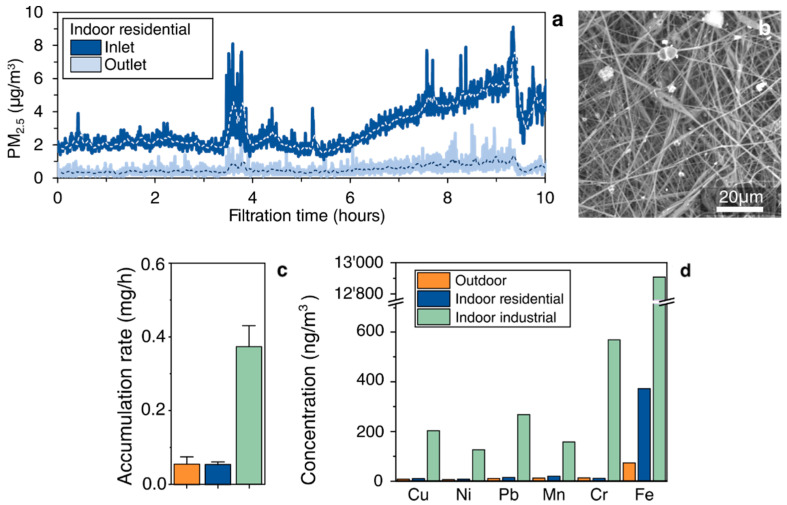
(**a**) Concentration of PM_2.5_ before and after 10 h of continuous single-PLA filtration; (**b**) SEM images of the electrospun mat after filtration; (**c**) accumulation of particulate over the filter, obtained by gravimetric analysis; (**d**) concentrations of metals in three different environments: outdoors, indoors residential, and indoors industrial.

Filtering activity was tested in different real environments, and the results are shown in Figure 3. The test was conducted by continuously monitoring the inlet and the outlet concentration of PM_2.5_ before and after the filter (Figure 4a), with the former taken as a reference value. The filtration efficiency was than confirmed by means of SEM images of the filters, where particles of particulate matter were evident on the surface of the filter (Figure 4b).

The outdoor and indoor residential concentrations were close to each other, showing the significant impact of outdoor pollutant sources on indoor levels [35]. In the case of indoors residential, there was no active air depuration or ventilation system. The highest accumulation rate was detected indoors, in the industrial scenario (Figure 4d). In this case, an active mobile local exhaust ventilation system was present for the control of welding fumes during working procedures, but the level of pollutants was three time higher than that measured both indoors and outdoors. It has been described that frequent changes in location and welding position make it more difficult to control fume exposure compared to industries where fixed locations are the norm; thus, the implementation of personal protective equipment (PPE) can lower the exposure workers [36].

In the case of mass profiles for indoors residential, metals exhibited similar values to outdoors, as also found by a few authors [37,38] in an indoor school environment (Figure 4e). In this case, as expected, the highest percentages of metals were also detected for indoors industrial. Fe, Cr, and Pb had the highest enhancement compared to the values obtained for indoors residential and/or outdoors.

## 4. Conclusions

An open-source electrospinning apparatus was designed, realized by 3D printing, and made available for preparing electrospun filter media. A solution of 14 wt.% PLA was spun over a 3D-printed scaffold, in order to obtain an effective, flexible, self-sustaining composite. 3D printing can provide, in emergency situations, a possible solution to produce, in a delocalized way, a sustainable alternative to PPE and to give competitive advantages. The necessity of more sustainable end-of-life solutions for PPE is also becoming a big challenge for the environment. The utilization of ecofriendly materials such as biodegradable polymers, and the use of inexpensive production apparatus contribute to sustainable development under the principle of a circular economy. According to filtration tests, the 3D-printed/electrospun composite exhibited a filtration efficiency more than 60%, comparable to the surgical mask (for the single filter), whereas the double-layered electrospun PLA revealed similar performance to the FFP2 mask, retaining up to 95% of the particles with a diameter greater than 230 nm. Prolonged filtration tests, in both the residential and the industrial scenario, proved the filter’s reliability and its possible use as PPE.

## Figures and Tables

**Table 1:** Brief literature review of PLA-based electrospun fibers.

Fiber Composition	Experimental Parameters (Rate, Solvent, App. Potential, Distance)	Fiber Diameter	Pressure Drop	Filtration Efficiency	Refs.
PLA/CyD	0.5 mL/h, 8% *w*/*v* DCM/DMF (8:2), 16 kV, 10 cm	350–990 nm	24–34 Pa	97% (PM_2.5_, incense burning)	[15]
PLA	1 mL/h, 10 wt.%, a DCM/DMF (7:3) solvent +15 (needle) −3 kV (collector)	~500 nm	96–102 Pa (8 cm·s^−1^)	96.22–99.1% (PM_2.5_, NaCl)	[24]
PLA—PLA/chain extender	DCM/DMF/TFE	From 3 µm to 270 nm	50–1000 Pa (20–948 L/m^−2^·s^−1^)	92.7–99% Clay (12.07 μm) carbon black (235 nm) TiO_2_ (95.3 nm)	[25]
PLA/cotton fabric layer	DCM Needleless electrospinning collector, −30 kV, rotating electrode 60 kV	8 ± 0.2 µm	35.78 Pa/cm^2^ (8 L/min^−1^)	97.9% (*S. aureus*)	[26]
PLA/PSQ	DMF/DCM (3:7 vol) 16 kV, 30 °C, 35~50% (RH), 8–20 cm	Average value from 400 nm to 580 nm	21.43–37.85 Pa/μm (5.3 cm·s^−1^)	>99.98% NaCl (0.3 μm)	[27]
PLA/montmorillonite/keratin	CHL/AC (70/30 *v*/*v*) and formic Acid, 19 kV, 15 cm, and 5 μL/min,	125–171 nm	2.88–107.92 Pa (0.12–0.24 m·s^−1^)	Not evaluated	[28]
PLA	CHL/DMSO 15 kV, 1.2 mL/h, 10 cm	40–100 nm	Not evaluated	Not evaluated	[29]
PLA/chitosan	DCM/DMAC 18 kV, collector rotating at 5.5 m/min, 14 cm	910–1500 nm	148 Pa (14 cm·s^−1^)	98.99% (NaCl) 99.4% (*E. coli*) 99.5% (*S. aureus*)	[30]

PLA poly(lactic acid); CyD: cyclodextrins; DMC: dimethyl carbonate; DMF: *N*,*N*-dimethylformamide; NaCl: sodium chloride; TFE: 2,2,2-trifluoroethanol; TiO_2_: titanium dioxide; DCM: dichloromethane; PSQ: poly(epoxy-amino) silsesquioxane; RH: relative humidity; CHL: chloroform; AC: acetone; DMSO: dimethyl sulfoxide; DMAC: dimethylacetamide; *E. coli*: *Escherichia coli*; *S. aureus*: *Staphylococcus aureus*.

## Data Availability

The electrospinning setup design files are available online at GitHub (https://github.com/piermatt/openspinnr, accessed on 5 November 2021). Raw data about the study can be provided upon email request.

## Supplementary Materials

The following are available online at https://www.mdpi.com/article/10.3390/ma14226766/s1: Table S1. BOM of the electrospinning setup; Figure S1. 3D-printed parts in electrospinning setup. (a) Collector ends and supports; (b) Slider and slider support; (c) Controller box; (d) Gear; Figure S2. Electronic schematic of electrospinning setup; Figure S3. (a) Schematic representation of the filtration setup test; (b) preparation of the sample (double-PLA) for the filtration tests; Figure S4. EM/EDX of the filter after 48 h filtration (outdoors).
