# Supplementary material for: Characterization and Filtration Efficiency of Sustainable PLA Fibers Obtained via a Hybrid 3D-Printed/Electrospinning Technique
Source: Materials (Basel). 2021 Nov 10;14(22):6766. doi: 10.3390/ma14226766 (PMC8624897; doi:10.3390/ma14226766)
Supplement: Supplementary file 1 [file materials-14-06766-s001.zip › materials-1343458-supplementary.pdf]

materials

MDPI

Supplementary Materials

Characterization and Filtration Efficiency of Sustainable PLA Fibers Obtained via a Hybrid 3D-Printed/Electrospinning Technique

Mattia Pierpaoli [1,*], Chiara Giosuè [2,*], Natalia Czerwińska [2], Michał Rycewicz [1], Aleksandra Wieloszyńska [1], Robert Bogdanowicz [1] and Maria Letizia Ruello [2]

1 Department of Metrology and Optoelectronics, Faculty of Electronics, Telecommunication and Informatics, Gdańsk University of Technology, 80-233 Gdańsk , Poland; michal.rycewicz@pg.edu.pl (M.R.); aleksandra.wieloszynska@pg.edu.pl (A.W.); robbogda@pg.edu.pl (R.B.)
2 INSTM Research Unit, Department of Materials, Environmental Sciences and Urban Planning (SIMAU), Università Politecnica Delle Marche, 60131 Ancona, Italy; n.czerwinska@pm.univpm.it (N.C.); m.l.ruello@univpm.it (M.L.R.)
* Correspondence: mattia.pierpaoli@pg.edu.pl (M.P.); c.giosue@univpm.it (C.G.)

Table S1. BOM of the electrospinning setup.

Component	Quantity	Unitary Cost ($)	Total Cost ($)	Material Type
Arduino nano board	1	2.07	2.07	Electronic
Rotary encoder	1	1.97 per 5 pcs	1.97	Electronic/Mechanical
DC-DC 5Vconverter	1	0.75	0.75	Electronic
0.91″ Oled display	1	1.4	1.4	Electronic
N20 Gear motor	1	1.35	1.45	Electronic/Mechanical
Plug	1	0.93	0.93	Electronic
Power supply (5 A, 12 V)	1	7.98	7.98	Electronic
High voltage transformer	1	3.86	3.86	Electronic
PWM module	1	0.97	0.97	Electronic
Ball bearing	1	1.58 per 10 pcs	1.58	Mechanical
28BYJ-48 5 V DC stepper motor with ULN2003 driver	2	1.85	3.7	Electronic/Mechanical
Split pin	1	5.49 per 50 pcs	5.49	Mechanical
M5 Threaded bar	40 cm	0.62	0.62	Mechanical
M5 nut	1	9.29 per 50 pcs	9.29	Mechanical
Plastic M6 rod	80 cm			
Dupont cable	1	1.58 per 40 pcs	1.58	Electronic
M3 nut	16	8.29 per 50 pcs	8.29	Mechanical
M3 bolt	16	0.84 per 20 pcs	0.84	Mechanical
Magnet	1	2.92 per 10 pcs	2.92	Mechanical

Switch	1	1.8 per 5 pcs	0.36	Electronic
3D printed parts	-	-	-	-

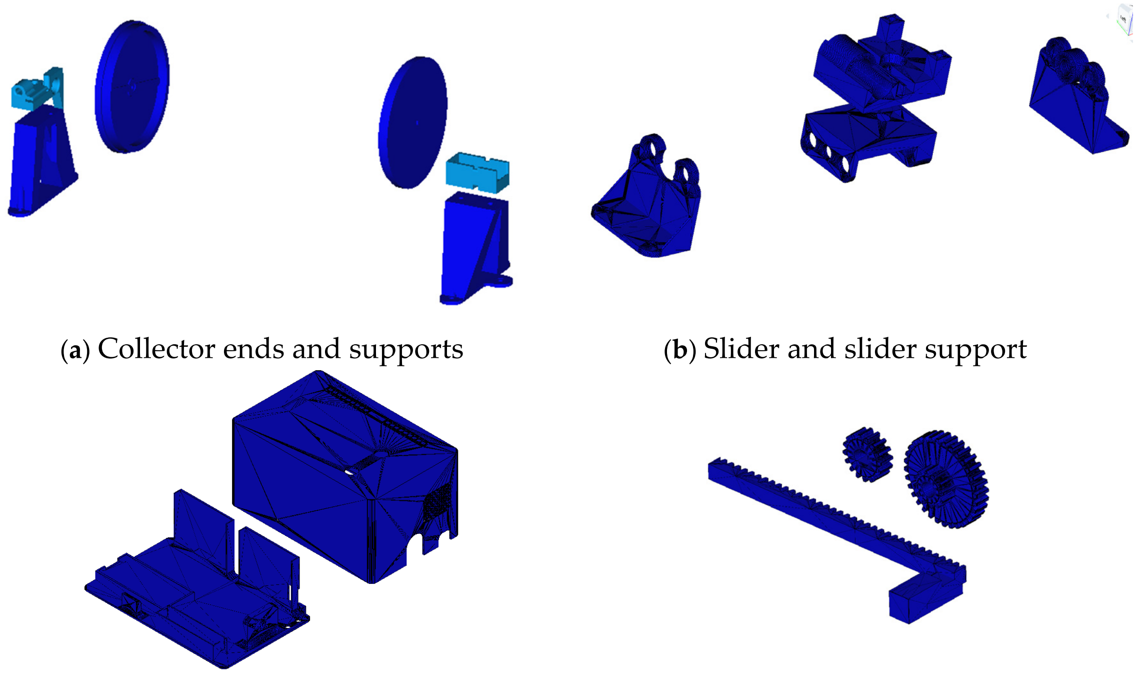

(**a**) Collector ends and supports
(**b**) Slider and slider support
(**c**) Controller box
(**d**) Gear

Figure S1. 3D-printed parts in electrospinning setup. (**a**) Collector ends and supports; (**b**) Slider and slider support; (**c**) Controller box; (**d**) Gear.

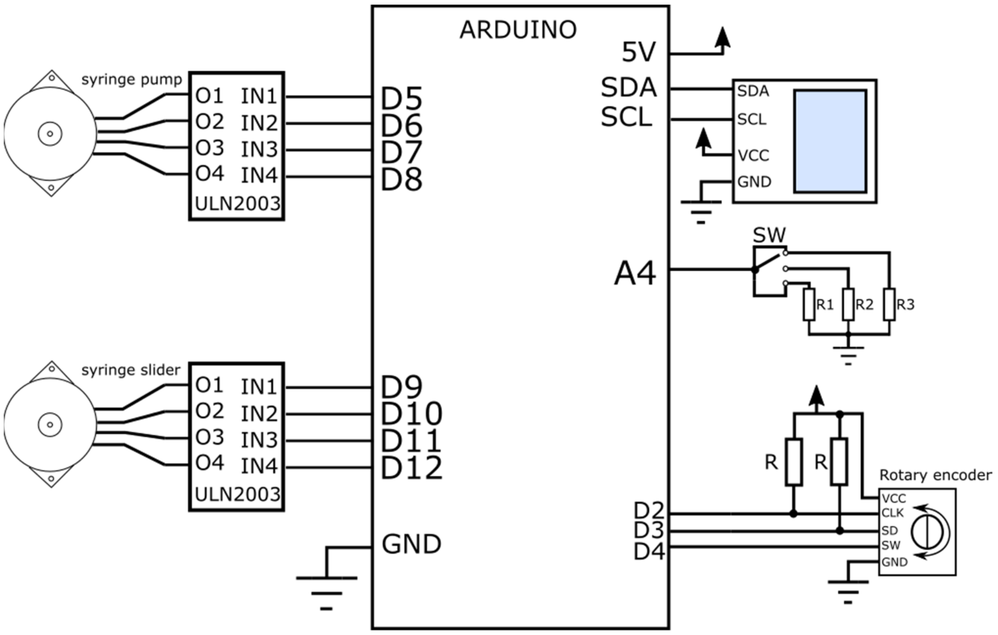

Figure S2. Electronic schematic of electrospinning setup.

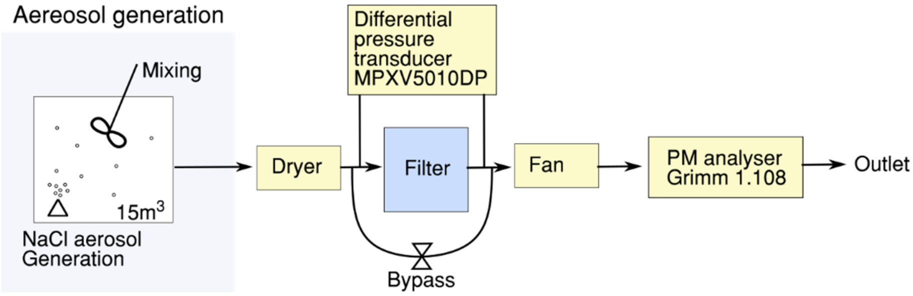

(**a**)

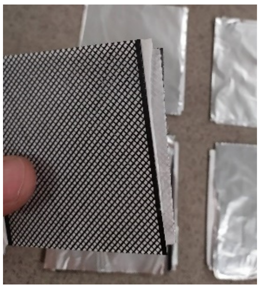

(**b**)

Figure S3. (**a**) Schematic representation of the filtration setup test (**b**) preparation of the sample (double-PLA) for the filtration tests.

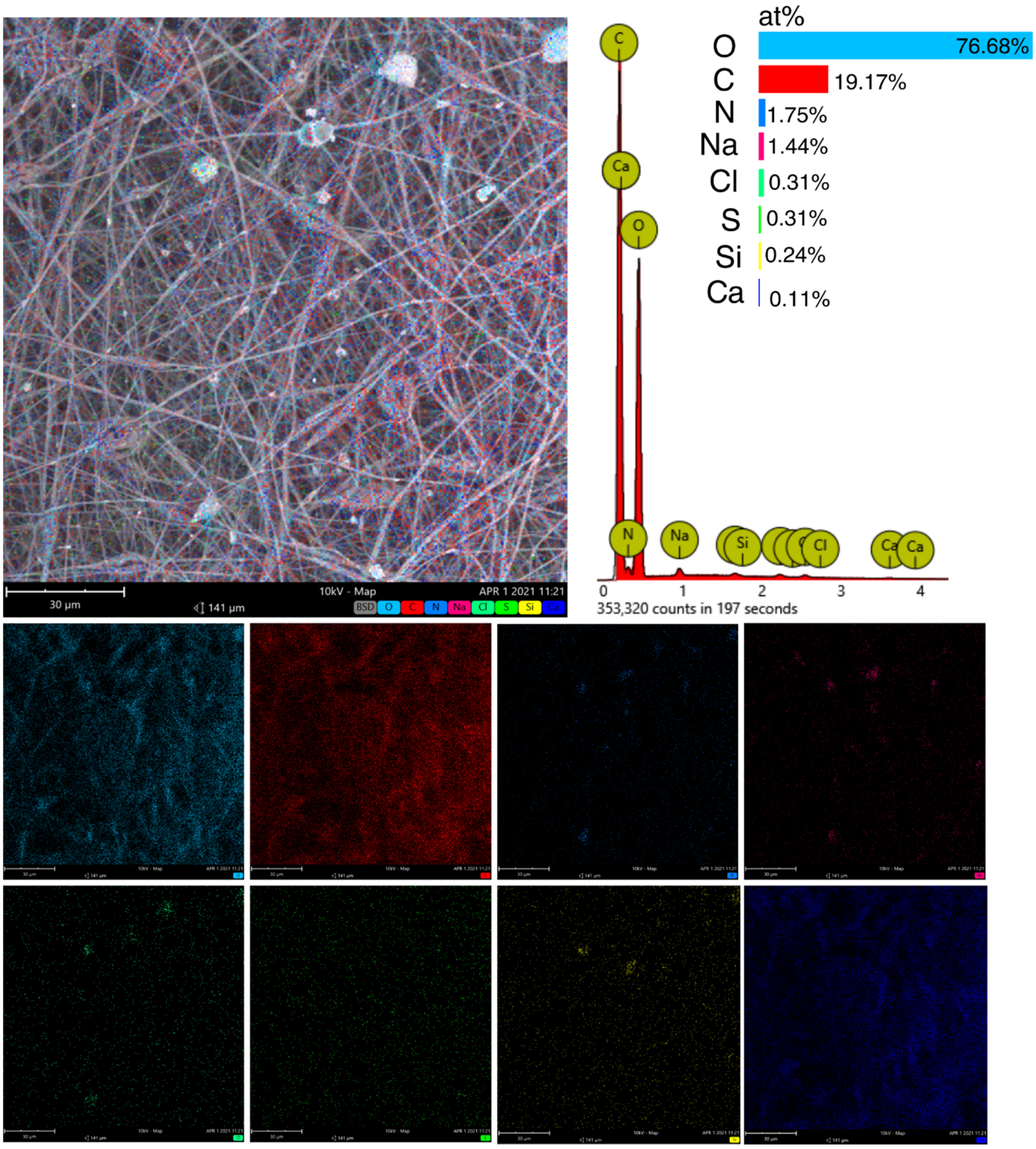

Figure S4. SEM/EDX of the filter after 48h filtration (outdoor).
